# Analgesic effects of oral Yokukansan on acute postoperative pain and involvement of the serotonin nervous system: a mouse model study

**DOI:** 10.1186/s12906-024-04501-6

**Published:** 2024-05-21

**Authors:** Shuichiro Kurita, Mika Sasaki, Moegi Tanaka, Yoshinori Kuwabara, Yukino Ogasawara, Hiroshi Baba, Yoshinori Kamiya

**Affiliations:** 1 Division of Anesthesiology, Niigata University Graduate School of Medical and Dental Sciences, 1-757 Asahimachi-dori, Chuo ward, Niigata, 951-8510 Japan; 2 Department of Palliative Care, Niigata City General Hospital, 463-7 Shumoku, Chuo ward, Niigata, 950-1197 Japan; 3 Department of Anesthesiology and Pain Medicine, Gifu University Graduate School of Medicine, Yanagido 1-1, Gifu, 501-1194 Japan; 4 Division of Kampo Pharmaceutical Sciences, Nihon Pharmaceutical University, 10281 Komuro, Ina, 362-0806 Japan

**Keywords:** Analgesia, Plantar incision model, Postoperative pain, Serotonin, Yokukansan

## Abstract

**Background:**

Yokukansan, a traditional Japanese medicine (Kampo), has been widely used to treat neurosis, dementia, and chronic pain. Previous in vitro studies have suggested that Yokukansan acts as a partial agonist of the 5-HT_1A_ receptor, resulting in amelioration of chronic pain through inhibition of nociceptive neuronal activity. However, its effectiveness for treating postoperative pain remains unknown, although its analgesic mechanism of action has been suggested to involve serotonin and glutamatergic neurotransmission. This study aimed to investigate the effect of Yokukansan on postoperative pain in an animal model.

**Methods:**

A mouse model of postoperative pain was created by plantar incision, and Yokukansan was administered orally the day after paw incision. Pain thresholds for mechanical and heat stimuli were examined in a behavioral experiment. In addition, to clarify the involvement of the serotonergic nervous system, we examined the analgesic effects of Yokukansan in mice that were serotonin-depleted by *para*-chlorophenylalanine (PCPA) treatment and intrathecal administration of NAN-190, 5-HT_1A_ receptor antagonist.

**Results:**

Orally administered Yokukansan increased the pain threshold dose-dependent in postoperative pain model mice. Pretreatment of *para*-chlorophenylalanine dramatically suppressed serotonin immunoreactivity in the spinal dorsal horn without changing the pain threshold after the paw incision. The analgesic effect of Yokukansan tended to be attenuated by *para*-chlorophenylalanine pretreatment and significantly attenuated by intrathecal administration of 2.5 µg of NAN-190 compared to that in postoperative pain model mice without *para*-chlorophenylalanine treatment and NAN-190 administration.

**Conclusion:**

This study demonstrated that oral administration of Yokukansan has acute analgesic effects in postoperative pain model mice. Behavioral experiments using serotonin-depleted mice and mice intrathecally administered with a 5-HT_1A_ receptor antagonist suggested that Yokukansan acts as an agonist at the 5-HT_1A_ receptor, one of the serotonin receptors, to produce analgesia.

## Introduction

Appropriate postoperative pain management is essential because ineffective management with incorrect opioid prescriptions can lead to opioid dependency [1] and delirium [2].

Pain has a complex relationship with the serotonergic signaling system: depending on the location and type of receptor expressed, serotonin can cause analgesia or hyperalgesia [3]. Although there is consensus that injury of nerves or the spinal cord directly leads to an increase in 5-HT in the injured tissue, data on remote effects in the nervous system remains controversial [4]. Inhibition of the serotonergic descending pain facilitatory pathway has been reported to have an analgesic effect. For example, inhibiting the 5-HT_3_ receptor with ondansetron reduces human neuropathic pain [5]. On the other hand, a previous study showed that activation of the spinal 5-HT_1A_ receptor alleviated acute pain in rats [6], suggesting that the serotonergic pain inhibitory pathway may contribute to antinociception in acute pain.

Yokukansan (YKS) is a traditional Japanese (Kampo) medicine used to treat sleep disorders and peripheral symptoms of dementia, such as increased aggression [7]. YKS is thought to exert its effects on the serotonergic neurotransmitter-mediated anxiolytic system through 5-HT_1A_ agonist action [8] and anti-neuroexcitatory activity via suppression of 5-HT_2A_ receptor expression [9] and the glutamatergic neurotransmitter-mediated system [10]. Clinical studies on the analgesic effects of YKS have suggested its usefulness in neuropathic pain [11]. A recent clinical study on acute postoperative pain after breast cancer surgery also demonstrated the perioperative anxiolytic effects of YKS [12]. Furthermore, although reviews have described the mechanism of action of YKS in neuropathic and chronic pain [13], no reports on its action on acute postoperative pain have been presented. However, since YKS has 5-HT_1A_ receptor agonist activity, we speculated that YKS would also exert an analgesic effect on acute pain, such as postoperative pain.

Thus, this study aimed to investigate the effect of YKS on postoperative pain. We hypothesized that YKS could reduce not only chronic pain but also acute postoperative pain and that this effect may be mediated by modulation of the serotonergic nervous system and descending inhibitory pathways. We created a mouse model of postoperative pain (modified from Brennan’s rat plantar incision model [14]) and conducted behavioral experiments to examine the analgesic effects of YKS.

## Methods

### Animals

The study is reported in accordance with ARRIVE guidelines. All animal experiments were approved by the Niigata University Ethics Committee for Animal Experiments (SA01010) and conducted in accordance with the guidelines of the Science Council of Japan.

C57BL/6 J mature male mice (7–8 weeks old, 24–27 g/body) were obtained from Asazuma Animal Medical Instruments (Niigata City, Japan). The mice were housed in groups of 1–5 mice per cage under an environment with a stable temperature of 25 °C, a 12-h light/dark cycle, and free access to water and food.

### Drugs and administration

Yokukansan (Yokukansan extract granules; YKS, TJ-54, Lot No. 2110054010, Tsumura & Co. Tokyo, Japan), which is manufactured and provided by Tsumura & Co. and approved for ethical use by the Ministry of Health, Labor, and Welfare of Japan, is a dried extract of the following botanical raw materials: Atractylodes lancea rhizome, Poria sclerotium, Cnidium rhizome, Uncaria hook, Japanese Angelica root, Bupleurum root, Glycyrrhiza (Table 1). The seven medical herbs were extracted with purified water at 95˚C for 1 h, and the extract solution was separated from the insoluble waste and concentrated by removing water under reduced pressure. Spray drying was used to produce a dried extract powder. Seven and a half grams of YKS contains 3.25 g of a dried extract mentioned above and inactive ingredients (lactose hydrate and magnesium stearate). For YKS treatment, a dry powder extract of YKS was suspended in 0.25 ml distilled water. YKS was administered by oral gavage using a sonde (KN-348, Natsume Seisakusho, Tokyo, Japan) at doses of 0.25, 0.5, and 1.0 g/kg. The Doses of YKS were determined based on a previous study [15] and according to the estimated ED50, for heat stimuli based on the dose–response relationship of the analgesic effect of YKS on thermal stimuli in postoperative pain model mice (Fig. 2). Moreover, administration of YKS 2.0 g/kg induced a sedative state in naïve mice in the preliminary experiment, and we decided the maximal dose of YKS as 1.0 g/kg. The same volume of distilled water (0.25 ml) was administered orally as a control.

**Table 1:** Scientific species names of all ingredients of Yokukansan

Botanical raw materials	Scientific species name	Part	Proportion
Atractylodes lancea rhizome	*Atractylodes lancea*	rhizome	4
Poria sclerotium	*Poria cocos*	sclerotium	4
Cnidium rhizome	*Cnidium officinale*	rhizome	3
Uncaria hook	*Uncaria rhynchophylla*	thorn	3
Japanese Angelica root	*Angelica acutiloba*	root	3
Bupleurum root	*Bupleurum falcatum*	root	2
Glycyrrhizae radix	*Glycyrrhiza uralensis*	root and stolon	1.5

*Para*-Chlorophenylalanine (PCPA, R&D Systems, Inc. Minneapolis, USA) depletes serotonin (5-hydroxytryptamine, 5-HT) by inhibiting tryptophan hydroxylase, a serotonin synthesis enzyme [16]. To deplete serotonin, the mice were treated with PCPA (150 mg/kg/day, diluted with distilled water) administered subcutaneously for 7 days under anesthesia (the same anesthetic method used to create the postoperative pain model) via an osmotic pump (Cat# 1007D, Alzet Osmotic Pumps, Cupatino, CA, USA) surgically implanted subcutaneously in the back of the mice. The control group received the same volume of distilled water as the PCPA-administered group. Mice receiving PCPA and distilled water were each marked with an individual-colored marker and kept in mixed conditions.

1-(2-methoxyphenyl)-4-(4-[2-phthalimido]butyl) piperazine hydrobromide (NAN-190, 5-HT_1A_ receptor antagonist, 0.5, 2.5 µg/body; MedChemExpress, Monmouth Junction, NJ, USA) was dissolved in 10% dimethyl sulfoxide (DMSO, Wako Pure Chemicals, corp., Osaka, Japan) diluted with saline. The dose of NAN-190 utilized was selected based on previously studies [17, 18].

The intrathecal injection procedure was adapted from the method of Hylden and Wilcoxon with some modification [19, 20]. Briefly, the lumbar puncture was performed using a 30-gauge needle attached to a 50-µl Hamilton microsyringe. The needle was inserted between L5 and L6, and drugs were delivered in a volume of 5 ml in conscious mice. The mice were not anesthetized during these procedures. Puncture of the dura was behaviorally indicated by a slight flick of the tail. Intrathecal administration of 10% DMSO and NAN-190 was performed 15 min before oral administration of YKS 0.5 g/kg.

### Surgical protocol

A modified version of Brennan’s procedure, established in a previous study [14], was used to create a mouse model of postoperative pain. All mice were anesthetized with 3% isoflurane administered through a nose cone, and the left plantar region was disinfected with 10% povidone–iodine solution. A 5-mm incision was made in the left hind paw, starting 2 mm from the proximal edge of the heel. The underlying flexor muscle was elevated and transversely incised. The surgical wound was closed by suturing the skin at the incision site at two places using a 7–0 nylon thread. Sham mice were only disinfected under the same anesthesia as the model mice, without incision.

### Study groups

The following groups were used to demonstrate the effects of YKS on postoperative pain: 1) the B group (Brennan’s plantar incision), which received a plantar incision and were the postoperative pain model mice; 2) the BY group, which received YKS after plantar incision. To confirm that the effects of YKS were mediated by the serotonergic nervous system, we prepared the following groups: 3) the PB group, which pretreated *para*-chlorophenylalanine (PCPA), to deplete serotonin, and plantar incision; 4) the PY group, which received PCPA and YKS; and 5) the PBY group, which received PCPA, plantar incision, and YKS. To determine whether the antinociceptive effect of oral YKS was involved in activation of 5HT_1_, we prepared the following groups: 6) the B (intrathecal 10% DMSO) group, which received intrathecal 10% DMSO and plantar incision; 7) the BN group, which received intrathecal NAN-190 (2.5 µg/body) and plantar incision; 8) the BY (intrathecal 10% DMSO) group, which received intrathecal 10% DMSO, plantar incision and YKS; 9) the BNY group, which received intrathecal NAN-190, plantar incision and YKS.

### Behavioral experiments

Behavioral experiments were performed by assessors blinded to the drug administration in the soundproof room. To avoid affecting circadian rhythms, all behavioral experiments were conducted at 9:00 AM, and the animals were allowed to acclimatize for at least 30 min. The von Frey (mechanical stimulation test) [21] and Hargreaves (thermal stimulation test) [22] tests were used to evaluate the paw-withdrawal threshold. For the mechanical stimulation test, the mice were placed in a red transparent plastic chamber on a wire grid and the paw-withdrawal threshold of the mechanical stimuli was measured. The Semmes–Weinstein set of monofilaments (von Frey filaments) was used to stimulate the central part of the left plantar region. The paw-withdrawal threshold was defined as the value of the smallest filament that elicited withdrawal reflexes in two of 10 stimulations.

For the heat stimulation test, the mice were placed in a red transparent plastic chamber on a wire grid and allowed to acclimate for at least 30 min. Paw-withdrawal latency induced by heat stimulation was measured. The left hind paw was irradiated using radiant heat (Hargreaves apparatus model 7370, Ugo Basile, Comerio, Italy). The average of three measurements taken at 3–5-min intervals was used as the latency to escape pain. Both heat and mechanical stimulations were performed immediately before YKS/distilled water administration and 30, 60, 90, 120, 180, 240 and 480 min after administration. The heat stimulation test for PCPA-treated mice was started before PCPA was administered via the osmotic pump (for control), immediately before plantar incision on the day before the heat stimulation test, and continued for an 8-h period as well. The heat stimulation test for intrathecal NAN-190 mice was same as main behavioral experiment up to 240 min after YKS administration.

### Immunostaining

The mice were euthanized by isoflurane anesthesia inhalation overdose. Immediately, 25 mL of saline followed by an equal amount of 4% paraformaldehyde (Mildform® 10N, Fujifilm Wako Pure Chemical Company, Osaka, Japan) were injected via transcardial perfusion. Each lumbar enlargement of spinal cord was resected and post-fixed at 4 °C overnight in the 4% paraformaldehyde and subsequently equilibrated in 20% sucrose in 0.1 M phosphate-buffered saline (PBS) overnight at 4 °C for cryoprotection. Spinal cord tissue was embedded in FSC22 (frozen-section medium; Leica Biosystems, Wetzlar, Germany) and frozen at -70 °C until sectioning. Spinal cord Sects. (10 µm) were prepared on a frozen microtome (CM1520, Leica Biosystems) at -20 °C, mounted on glass slides (Matsunami GlassInd. Ltd., Osaka, Japan) and stored at -70 °C.

The sections were washed twice with TNT buffer (10% 1 M Tris–HCl, pH 7.5, 5% 3 M NaCl, 0.03% Tween 20) and incubated with Blocking One Histo (Nacalai tesque, Kyoto, Japan) at room temperature (23–25 °C) for 1 h. After removing the blocking buffer, the sections were incubated in a humidified chamber at 4 °C for 2 days with rabbit anti-5-HT (Serotonin) IgG (1:20,000; Cat. #; 20,080, Immunostar, Wisconsin, USA), which were diluted with 0.1% Tween 20 in TNB buffer (0.1 M Tris–HCl buffered saline, pH 7.5, containing 1% blocking reagent. Sections were then rinsed twice with TNT buffer and incubated overnight in a humidified chamber at 4 °C with Cy3-conjugated goat anti-rabbit IgG (1:1000; Cat. #; 111–167-003, Jackson ImmunoResearch Laboratories, Inc., West Grove, PA) for anti-5-HT IgG. The sections were washed twice with TNT buffer, embedded using VECTASHIELD Antifade Mounting Medium (Vector Laboratories Inc., Newark, CA, USA), and visualized using a fluorescence microscope (BX 53, Olympus, Tokyo, Japan) equipped with a digital camera system (DP73, Olympus, Tokyo, Japan). We analyzed the staining results using BZ-H4C analyzer software (Keyence). The area considered to be layers I and II in the spinal cord dorsal horn image taken at × 20 was enclosed, and the points within it, stained using serotonin, were counted using a hybrid cell count function [23].

### Statistical analysis

The sample size was determined based on previous behavioral and immunohistological studies. We did not calculate statistical power a priori. A post-hoc power analysis was conducted using G* Power version 3.1.7 [24], which indicated that a sample of five to six mice/group for behavioral experiments was sufficient to reach statistical significance with power (1—β) set at 0.8 and α = 0.05. Data are presented as mean ± standard error of the mean.

The dose–response relationship of YKS to mechanical and heat stimuli was fitted using GraphPad Prism 9 software (GraphPad Software, La Jolla, CA, USA) according to the following equation:

$$\mathrm Y=\frac{\mathrm{maximal}\;\mathrm{analgesic}\;\mathrm{effect}\;(\mathrm{set}\;\mathrm{as}\;100\%)}{1\;+\;{(\mathrm D/{\mathrm{EC}}_{50})}^{\mathrm n}}$$

where Y is the relative analgesic effect normalized to the maximal analgesic effect, D is the dose of YKS (mg/kg), n is the Hill coefficient, and EC_50_ is the half-maximal effective concentration for analgesic effect of YKS.

We used repeated-measures (RM) two-way analysis of variance (ANOVA) for behavioral data after tests of homoscedasticity and normality of the datasets using the Shapiro–Wilk test. Repeated-measures analyses were performed without sphericity assumption, and the Greenhouse– Geisser correction was applied. If the RM two-way ANOVA revealed statistical significance, multiple comparisons using Dunnett's test with reference to B groups were performed. For experiments using PCPA pretreated mice, Bonferroni’s multiple comparison test was applied. Immunohistological quantification in dorsal horn of spinal cord was analyzed using one-way ANOVA followed by Tukey’s test. All statistical analyses were performed using GraphPad Prism10 for Mac software. Statistical significance was set at *P* < 0.05.

## Results

### Effects of oral Yokukansan on postoperative pain

**Fig. 1:**
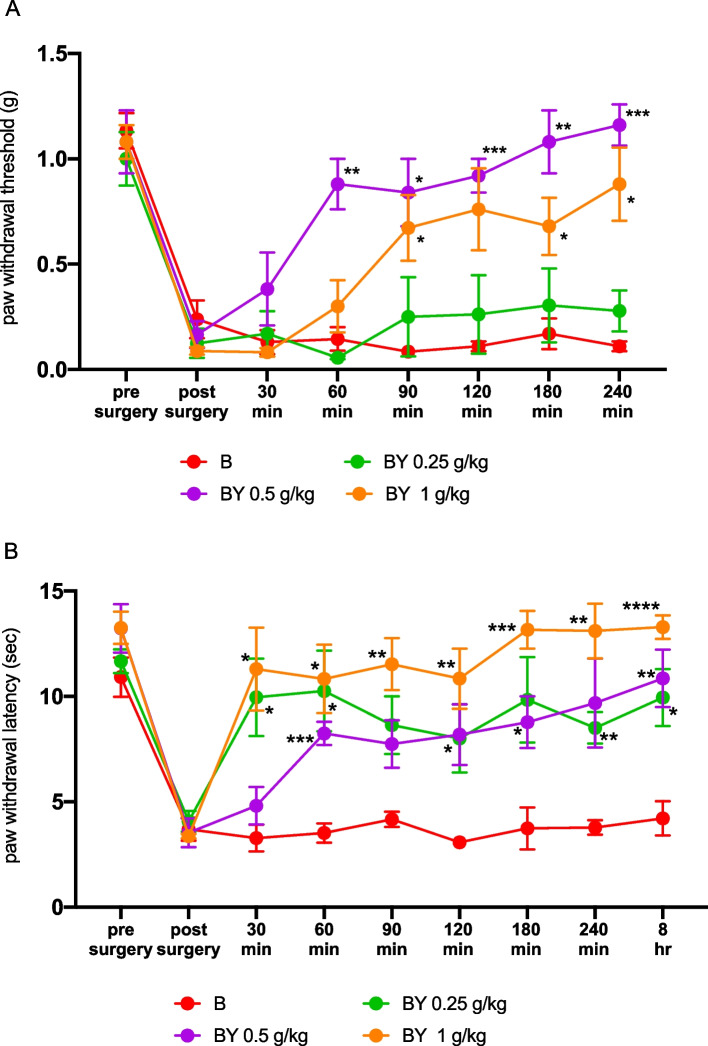
Time-course plots showing changes in paw-withdrawal thresholds and latencies before and after oral administration of Yokukansan (YKS) in postoperative pain model mice. **A** Paw incision elicited significant decrease of paw-withdrawal thresholds by mechanical stimuli using von Frey hairs. In this model mice, orally administered YKS ameliorated paw-withdrawal threshold at 0.5 g/kg and 1 g/kg. **B** Paw incision also elicited a significant decrease in paw-withdrawal latencies in response to radiant heat stimuli. Orally administered YKS decreased paw-withdrawal latencies to mechanical stimulation similarly, at all doses (0.25–1.0 g/kg). * *P* < 0.05; ** *P* < 0.01; *** *P* < 0.001; **** *P* < 0.0001 compared with the B group by a repeated-measures two-way analysis of variance, followed by Dunnet’s test. B: Brennan model group; BY: Brennan model group treated with YKS; YKS: Yokukansan

The mechanical stimulation test showed no significant differences in the baseline thresholds between mice. However, at YKS doses of 0.5 mg/kg or higher, the BY group had significantly higher paw-withdrawal thresholds than did the B group, 60–240 min after YKS administration. (*n* = 6 for B group and *n* = 5 for each BY group; Time: F (4.283, 72.80) = 42.32, *P* < 0.0001; Group: F (3, 17) = 11.14, *P* = 0.0003; time × Group: F (21, 119) = 6.123, *P* < 0.0001 by repeated-measures two-way ANOVA, Fig. 1A). In the thermal stimulation test, paw withdrawal latency was significantly prolonged in the BY group compared to the B group at all doses from 60 min to 8 h after YKS administration (*n* = 6 for each group; Time: F (5.227, 104.5) = 19.05, *P* < 0.0001; Group: F (3, 20) = 18.08, *P* < 0.0001; Time × Group: F (24, 160) = 2.557, *P* = 0.0002 by RM two-way ANOVA, Fig. 1B). Interestingly, the anti-hyperalgesic effect of YKS was observed faster with thermal stimulation than with mechanical stimulation (Fig. 1A and B).

**Fig. 2:**
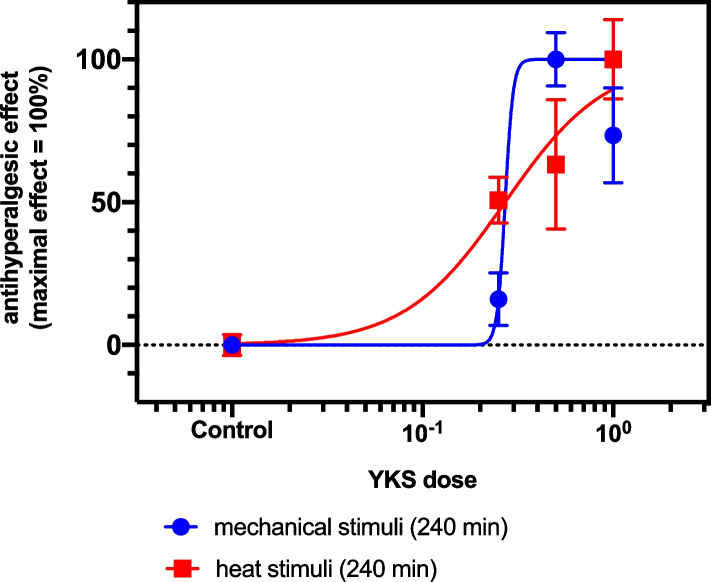
Dose–response relationship of the analgesic effect of orally administered Yokukansan (YKS) in response to mechanical and heat stimuli. The relative analgesic effects normalized to the maximal analgesic effect in paw-withdrawal threshold and latencies in response to mechanical and heat stimulation are shown in blue square and red circles, respectively. The solid lines in the graph represent the fit of the Hill equation. The YKS dose of maximal analgesic effects for mechanical and heat stimuli were 0.5 g/kg and 1 g/kg, respectively. The dose for 50% maximal analgesic effect (ED_50_) for heat stimuli was 0.27 g/kg (95% confidence interval; 0.087 to 0.447 g/kg) and Hill coefficient was 1.65 (95% confidence interval; 0.71 to 3.77). The dose–response relationship of analgesic effect of YKS to mechanical stimuli was very steep (ED50: 0.27 g/kg, Hill coefficient: 27.4). YKS: Yokukansan

The dose–response relationship of the analgesic effect of YKS was calculated using the aforementioned equation and estimated at 0.27 g/kg (95% confidence interval: 0.087–0.447 g/kg) for heat stimuli, whereas that for mechanical stimuli had a very high Hill coefficient and wide range of EC50 (Fig. 2). We therefore chose heat stimulation for evaluation of hyperalgesia and a dose of 0.5 g/kg YKS in PCPA-treated mice in subsequent experiments.

### Effect of oral Yokukansan on PCPA-treated mice

**Fig. 3:**
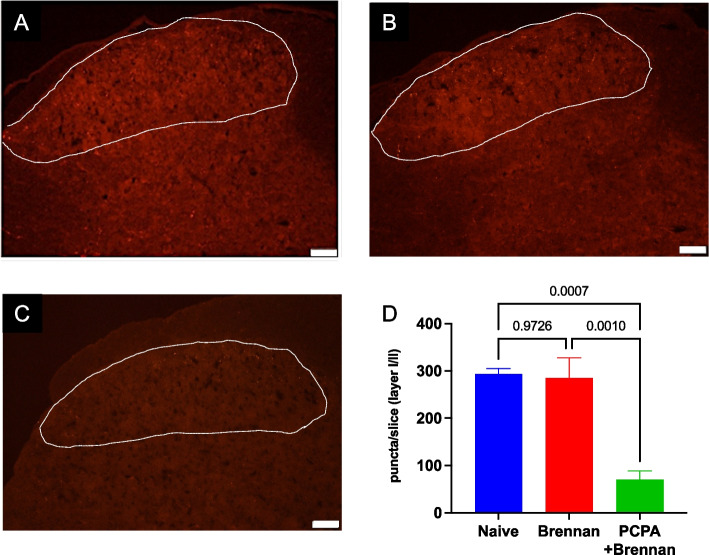
Paw injury did not affect serotonin expression in the spinal dorsal horn, and *para*-chlorophenylalanine (PCPA)-treatment dramatically reduced serotonin expression in the spinal dorsal horn. **A** Serotonin expression in the spinal dorsal horn was observed in normal mice. **B** Plantar incision did not affect serotonin expression in the spinal dorsal horn in the substantia gelatinosa (layer I to II of Rexed). **C** After PCPA administration (150 mg/kg/day, 7 days), serotonin expression in the spinal dorsal horn was dramatically reduced even after paw injury. Scale bar: 50 µm. The white dotted line indicates the region of interest of 5-HT-immunoreactive puncta counting. **D** Summary of quantification of serotonin-immunopositive puncta in the spinal dorsal horn. *N* = 4 for each group. Data are expressed as the mean ± SEM for each group and statistical analysis was performed using one-way ANOVA followed by Tukey’s test

Serotonin expression was observed in the spinal dorsal horn, particularly in the outer layer, of normal mice (Fig. 3A) and was not changed in paw-injured mice (Fig. 3B). PCPA administration for 7 days dramatically reduced serotonin expression in the spinal dorsal horn of paw-injured mice (Fig. 3C, D).

**Fig. 4:**
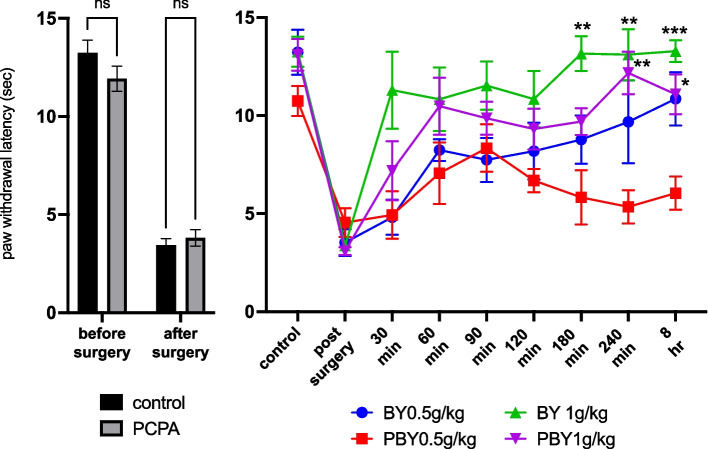
Analgesic effect of Yokukansan (YKS) against heat stimulation in *para*-chlorophenylalanine (PCPA)-treated, postoperative pain model mice. **A** The paw-withdrawal latencies were not influenced by PCPA treatment both before and after paw injury. **B** The analgesic effect of YKS 0.5 g/kg in postoperative pain model mice after PCPA treatment was attenuated than that in normal (PCPA-untreated) mice. However, YKS 1.0 g/kg had a significant analgesic effect and this effect was dose-dependent, even in PCPA-treated mice. * *P* < 0.05; ** *P* < 0.01; *** *P* < 0.001; **** *P* < 0.0001 compared to the PB group by a repeated-measures two-way analysis of variance, followed by Dunnet’s test. YKS: Yokukansan; PY: PCPA and YKS treated group; PB: Brennan model in PCPA-treated group; BY: Brennan model group treated with YKS; PBY: Brennan model in PCPA-treated group treated with YKS

Pretreatment with PCPA did not alter the threshold to mechanical stimuli compared with normal (PCPA-untreated) mice, nor did it change the mechanical hypersensitivity induced by plantar incision. (*n* = 12 for each group; Incision: F(1, 22) = 267.5, *P* < 0.0001; Treatment: F(1, 22) = 0.8567, *P* = 0.3647; Incision × Treatment: F(1, 22) = 2.358, *P* = 0.1389 by RM two-way ANOVA, Fig. 4A). In the PCPA-treated paw-injured (PBY) mice, the analgesic effect of YKS (0.5 g/kg) was attenuated as compared with the normal paw-injured (BY) mice. However, the analgesic effect of YKS was significantly greater in the PBY group at dose 1 g/kg than at dose 0.5 g/kg, especially 180 min after YKS administration (*n* = 5 for each group; Time: F (5.147, 102.9) = 22.51, *P* < 0.0001; Group: F (3, 20) = 10.56, *P* = 0.0002; Time × Group: F (24, 160) = 1.993, *P* = 0.0064 by repeated-measures two-way ANOVA, Fig. 4B).

### Effect of oral Yokukansan on intrathecally NAN-190 administered mice

A previous study suggested that YKS has a pharmacological effect due to its agonist action on 5-HT_1A_ receptors [8, 25, 26]. Based on this, we hypothesized that the analgesic effect of YKS may be attributed to the inhibition of 5-HT_1A_ receptors in the spinal cord. To investigate this, YKS was orally administered to model mice pretreated with the 5-HT_1A_ receptor antagonist NAN-190 via intrathecal administration. The effects of this treatment were examined to determine the analgesic effect for 240 min after oral administration of YKS 0.5 g/kg.

**Fig. 5:**
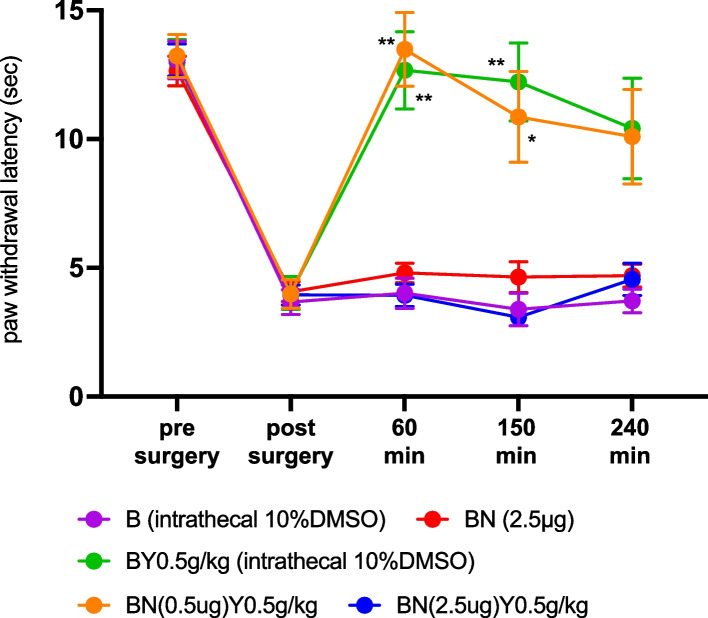
The effect of inhibition of 5-HT_1A_ receptor in the spinal cord against analgesic effect of oral Yokukansan (YKS) administration in postoperative pain model mice. Intrathecal administration of 10% DMSO containing normal saline and 2.5 µg/5µL of NAN-190, 5-HT_1A_ receptor selective antagonist. However, intrathecally administered 2.5 µg of NAN-190 completely abolished the analgesic effect of YKS 0.5 g/kg in postoperative pain model mice. A lower dose (0.5 µg) of NAN-190 did not block the analgesic effect of YKS 0.5 g/kg, suggesting that the analgesic effect of 5-HT1A inhibition in YKS analgesia may be dose-dependent. * *P* < 0.05; ** *P* < 0.01 compared to the B (intrathecal 10%DMSO) group by a repeated-measures two-way analysis of variance, followed by Dunnet’s test. YKS: Yokukansan; B (intrathecal 10%DMSO): Brennan model group that received 5µL of 10% DMSO containing normal saline intrathecally; BN (2.5 µg): Brennan model group treated with intrathecal NAN-190 (2.5 µg); BY0.5 g/kg (intrathecal 10%DMSO): Brennan model group that administered 5µL of intrathecal 10% DMSO containing normal saline and YKS; BN(0.5)Y0.5 g/kg: Brennan model group treated with intrathecal NAN-190 (0.5 µg) and YKS 0.5 g/kg; BN(2.5)Y0.5 g/kg: Brennan model group treated with intrathecal NAN-190 (2.5 µg) and YKS 0.5 g/kg

Intrathecal administration of 10% DMSO solution and 2.5 µg of NAN-190 was not influenced in paw withdrawal behavior of model mice. analgesic effect of oral administration of 0.5 g/kg YKS in the model mice was vanished by intrathecal administration of NAN-190 (2.5 µg/5µL). However, 0.5 µg of NAN-190 did not influenced analgesic effect of YKS 0.5 g/kg in the model mice (*n* = 7 for B (intrathecal10% DMSO and B (2.5 µg) group, *n* = 5 for BY0.5 g/kg (intrathecal 10% DMSO) and BN(0.5 µg)Y(0.5 g/kg) group and *n* = 10 for BN(2.5 µg)Y(0.5 g/kg) group; Time: F (3.356, 97.32) = 115.4, *P* < 0.0001; Group: F (4, 19) = 20.87, *P* < 0.0001; Time × Group: F (16, 116) = 10.74, *P* < 0.0001 by repeated-measures two-way ANOVA, Fig. 5).

## Discussion

This study demonstrated that YKS has analgesic effects on acute postoperative pain. We show that YKS administration in a dose dependent manner increased pain threshold in plantar incision pain model. We speculated that the serotonergic system was involved in the analgesic effect of YKS and thus evaluated paw-withdrawal latency in mice with serotonin-ablation after PCPA administration. Serotonergic ablation alone did not affect the paw-withdrawal latencies, with or without hind paw injury, suggesting that serotonin is not involved in acute pain perception. However, YKS showed a reduced analgesic effect in PCPA-treated mice. In contrast, when a higher dose of YKS was used, a more potent analgesic effect was observed in these mice, suggesting that YKS might act as an alternative to serotonin as an activator of the descending pain inhibitory system. Furthermore, intrathecal administration of the 5-HT_1A_ receptor antagonist NAN-190 suppressed the analgesic effect of YKS, suggesting that activation of the serotonin 5-HT_1A_ receptor is involved in the analgesic effect of YKS. Thus, this study demonstrates that YKS has an analgesic effect in a mouse model of postoperative pain and that the analgesic effect is derived from the direct activation of 5-HT_1A_ receptors in the spinal cord rather than from the activation of serotonergic pathways in the descending pain inhibitory system.

Serotonin is known to exert its analgesic effect by activating the descending pain inhibitory system, while at the same time acting as a pain-producing substance in its own right and as a neurotransmitter in the descending pain facilitatory system [3]. A recent study revealed that the serotonergic descending pain inhibitory system changes to pain facilitatory properties in neuropathic pain model mice due to a reduction in KCC2 ion transporter (K-Cl cotransporter) expression in the spinal dorsal horn neurons. In this way, serotonergic neuronal transmission is converted from being inhibitory to being excitatory [27]. However, acute pain due to tissue injury (intraplantar injection of formalin) reportedly stimulates serotonin secretion in the spinal cord more rapidly than does noradrenaline [28]. This finding indicated that serotonin may act as a pain-inhibitory neurotransmitter in the spinal cord during acute pain. In our study, it was shown that paw injury did not elicit serotonin expression in the spinal dorsal horn under immunohistochemical analysis. This result was consistent to the previous study that paw injury did not stimulate serotonin secretion in the spinal dorsal horn [29, 30]. Moreover, paw-withdrawal latencies in response to heat stimuli were not altered by paw injury, even in PCPA-induced serotonin-depleted mice, suggesting that serotonin does not exacerbate acute pain. Recently, gabapentinoid has been shown to activate the descending pain inhibitory system in both the noradrenergic and serotonergic pathways. Noradrenergic depletion quenched the analgesic effect of gabapentinoid [23], suggesting that serotonin does not play a major role in the descending pain inhibitory system even in a neuropathic pain state. This suggests that serotonin in the spinal dorsal horn is not a major modulator, even in acute pain such as postoperative pain.On the other hand, the analgesic effect of YKS is weakened in serotonin-depleted mice by PCPA administration, then it cannot be ruled out that YKS exerts an analgesic effect by activating the descending pain inhibitory system.

The roles of serotonin in nociception are complex. A previous study showed that the subcutaneous injection of serotonin induced hyperalgesic behaviors in rats via 5-HT_1A_ receptor activation [31]. In contrast, 5-HT_1A_ receptor activation elicits anti-nociception in both acute and chronic (including neuropathic) pain [32–34]. In addition, acute pain and inflammation induced by subcutaneous administration of serotonin are ameliorated by 5-HT_2A_ antagonists [35]. A previous in vitro study suggested that YKS acts as a partial agonist of the 5-HT_1A_ receptor [25] and that 5-HT_1A_ receptor activation induces a decrease in cAMP levels by activating G_i/o_, which may suppress neuronal activity. Moreover, YKS induces down-regulation of the 5HT_2A_ receptor in vivo [9]. The results of our study are consistent with those of previous studies on the effects of YKS on serotonergic receptors, which suggested that YKS acts as an antinociceptive agent and a serotonin agonist in mice with PCPA-mediated serotonin ablation, because YKS augmented paw-withdrawal latencies in a dose–response manner. Furthermore, we have shown that intrathecal administration of NAN-190, a 5-HT_1A_ receptor antagonist, prior to YKS administration dampened the analgesic effects of YKS, suggesting that the analgesic effect of YKS activates the 5-HT_1A_ receptor in the spinal cord. This evidence reinforces the concept that YKS acts as a 5-HT_1A_ receptor agonist.

### Limitations

This study had several limitations. First, we did not evaluate changes in 5HT_2A_ receptors in the spinal dorsal horn. However, a behavioral study indicated that YKS attenuated paw withdrawal latencies, even in serotonin-ablated mice, and YKS showed analgesic effects by affecting the serotonergic pathway. In addition, we did not evaluate the quantitative changes in serotonin expression in the spinal dorsal horn following paw injury or YKS administration. YKS includes *Uncaria rhynchophylla*, which has an indole skeleton. This serotonin-like structure may result in false-positive staining when using immunohistochemistry [36]. Further analysis using mass spectrometry is required to evaluate the effects of YKS on serotonin secretion.

Second, the serotonergic pathway affects mood, which may affect the pain threshold. YKS improves depressive behaviors in both humans [37, 38] and experimental animals [39, 40]. This may indicate that YKS acts as an antidepressant, in addition to its analgesic effects, particularly in neuropathic pain [41]. Further studies may be needed to evaluate the relationship between the potency of YKS as an antidepressant and its analgesic effects in experimental animals and humans.

## Conclusion

This study demonstrated that YKS has analgesic effects on postoperative pain and that the mechanism of analgesic action is derived from the direct activation of 5-HT1A receptors in the spinal cord and the activation of the serotonergic descending pain inhibitory system.

## Data Availability

The data produced and analyzed in this study are available from the corresponding author upon reasonable request.

## Abbreviations

5-HT: Serotonin
ANOVA: Analysis of variance
PCPA: *para*-Chlorophenylalanine
TNT: Tris–NaCl–Tween
TNT: Tris–NaCl–Tween buffer
YKS: Yokukansan
